# Age-associated defect in ADCC response to COVID-19 vaccine

**DOI:** 10.1038/s41541-025-01196-9

**Published:** 2025-07-01

**Authors:** Benjamin L. Sievers, Mazharul Altaf, Mark T. K. Cheng, Kimia Kamelian, Claire Cormie, The CITIID-NIHR BioResource COVID-19 Collaboration, John R. Bradley, Stephen Baker, Barbara Graves, Hannah Stark, Sabine Hein, Ingrid Scholtes, Daniela Caputo, Anne Elmer, Emma Le Gresley, Nathalie Kingston, Patrick Chinnery, Daniel Cooper, Gordon Dougan, Ian Goodfellow, Nathalie Kingston, Paul J. Lehner, Paul A. Lyons, Nicholas J. Matheson, Caroline Saunders, Kenneth G. C. Smith, Charlotte Summers, James Thaventhiran, M. Estee Torok, Mark R. Toshner, Michael P. Weekes, Gisele Alvio, Sharon Baker, Areti Bermperi, Karen Brookes, Isabel Cruz, Ranalie de Jesus, Katie Dempsey, Giovanni Di Stephano, Jason Domingo, Sarah Hewitt, Heather Jones, Sherly Jose, Jenny Kourampa, Caroline McMahon, Vivien Mendoza, Charmain Ocaya, Ciro Pasquale, Marlyn Perales, Carla Ribeiro, Vargas, Bensi Vergese, Laura Watson, Jieniean Worsley, Julie-Ann Zerrudo, Laura Bergamashi, Gräf, Kelvin Hunter, Federica Mescia, John Allison, Heather Biggs, Helen Butcher, Matt Chandler, Debbie Clapham-Riley, Eleanor Dewhurst, Christian Fernandez, Anita Furlong, Jennifer Gray, Tasmin Ivers, Rachel Linger, Mary Kasanicki, Rebecca King, Sarah Meloy, Alexei Moulton, Francesca Muldoon, Nigel Ovington, Sofia Papadia, Christopher J. Penkett, Isabel Phelan, Venkatesh Ranganath, Roxana Paraschiv, Abigail Sage, Jennifer Sambrook, Katherine Schon, Kathleen E. Stirrups, Paul Townsend, Neil Walker, Jennifer Webster, Petra Polgarova, Sarah L. Caddy, Laura G. Caller, Yasmin Chaudhry, Martin D. Curran, Theresa Feltwell, Stewart Fuller, Iliana Georgana, Grant Hall, William L. Hamilton, Myra Hosmillo, Charlotte J. Houldcroft, Rhys Izuagbe, Aminu S. Jahun, Fahad A. Khokhar, Anna G. Kovalenko, Luke W. Meredith, Surendra Parmar, Malte L. Pinckert, Anna Yakovleva, Emily C. Horner, Lucy Booth, Alexander Ferreira, Rebecca Boston, Robert Hughes, Juan Carlos Yam Puc, Nonantzin Beristain-Covarrubias, Maria Rust, Thevinya Gurugama, Lihinya Gurugama, Thomas Mulroney, Sarah Spencer, Zhaleh Hosseini, Kate Williamson, Rainer Doffinger, Ravindra K. Gupta

**Affiliations:** 1 Department of Medicine, University of Cambridge, Cambridge, UK; 2 Vaccine Research Center, National Institute of Allergy and Infectious Diseases (NIAID), National Institutes of Health (NIH), Bethesda, MD USA; 3 Cambridge Institute of Therapeutic Immunology & Infectious Disease (CITIID), Department of Medicine, University of Cambridge, Cambridge, UK; 4 School of Clinical Medicine, University of Cambridge, Cambridge, UK; 5 Cambridge University Hospitals NHS Foundation Trust, Hills Road, Cambridge, Cambridgeshire United Kingdom; 6 Africa Health Research Institute, Durban, South Africa; 7 NIHR BioResource, Cambridge University Hospitals NHS Foundation Trust, Cambridge Biomedical Campus, Cambridge, UK; 8 Cambridge Clinical Research Centre, Addenbrooke’s Hospital, Cambridge University Hospitals NHS Foundation Trust, Cambridge, UK; 9 Department of Clinical Neurosciences, School of Clinical Medicine, University of Cambridge, Cambridge Biomedical Campus, Cambridge, UK; 10 Medical Research Council Mitochondrial Biology Unit, Cambridge Biomedical Campus, Cambridge, UK; 11 Cambridge University Hospitals NHS Foundation Trust, Cambridge Biomedical Campus, Cambridge, UK; 12 Global and Tropical Health Division, Menzies School of Heath Research and Charles Darwin University, Darwin, NT Australia; 13 Department of Public Health and Primary Care, School of Clinical Medicine, University of Cambridge, Cambridge Biomedical Campus, Cambridge, UK; 14 Division of Virology, Department of Pathology, University of Cambridge, Cambridge, UK; 15 Department of Infectious Diseases, Addenbrooke’s Hospital, Cambridge University NHS Hospitals Foundation Trust, Cambridge, UK; 16 NHS Blood and Transplant, Cambridge, UK; 17 Intensive Care Unit, Addenbrooke’s Hospital, Cambridge University Hospitals NHS Foundation Trust, Cambridge Biomedical Campus, Cambridge, UK; 18 Heart and Lung Research Institute, Cambridge Biomedical Campus, Cambridge, UK; 19 MRC Toxicology Unit, Gleeson Building, Tennis Court Road, Cambridge, UK; 20 Department of Microbiology, Addenbrooke’s Hospital, Cambridge University NHS Hospitals Foundation Trust, Cambridge, UK; 21 Royal Papworth Hospital NHS Foundation Trust, Cambridge, UK; 22 Cambridge Institute for Medical Research, Biomedical Campus, Hills Rd, Cambridge, UK; 23 Clinical Genetics, Addenbrooke’s Hospital, Cambridge University Hospitals NHS Foundation Trust, Cambridge, UK; 24 University of Cambridge, Cambridge, UK; 25 The Francis Crick Institute, London, UK; 26 Public Health England, Clinical Microbiology and Public Health Laboratory, Cambridge, UK

**Keywords:** Immunology, Adaptive immunity, Vaccines

## Abstract

We investigated age-associated effects of SARS-CoV-2 vaccination in elderly individuals (n = 50, mean age 79) after six SARS-CoV-2 vaccine doses. While neutralization titers remained comparable across age groups, Fc-mediated effector functions declined with age. Individuals >80 demonstrated reduced antibody-dependent cellular cytotoxicity (ADCC), via a surrogate ADCC-signaling assay, correlating with diminished IgG1 binding. These findings highlight age-related impairments in Fc-mediated responses, with implications for immune protection and vaccine strategies in older populations.

## Aging and Fc-mediated effector functions

The SARS-CoV-2 pandemic has underscored the critical importance of effective vaccination strategies to mitigate severe disease and mortality, particularly among vulnerable populations such as the elderly^1–3^. Aging is associated with profound changes in the immune system, including diminished adaptive immune responses and alterations in innate immune function^4–6^. These changes, collectively termed “immunosenescence,” have been implicated in reduced vaccine efficacy and poorer outcomes in older individuals following viral infection^7–9^. Despite the widespread administration of COVID-19 vaccines, the extent to which aging influences the immune responses elicited by vaccination remains incompletely understood^10–13^.

Neutralizing antibodies targeting the SARS-CoV-2 spike protein are a central component of vaccine-induced immunity, preventing viral entry and replication^14–16^. However, the role of Fc-mediated effector functions, such as antibody-dependent cellular cytotoxicity (ADCC), in vaccine protection has garnered increasing attention during the pandemic^17–19^. These effector functions, mediated through the interaction of antibody Fc regions with Fc gamma receptors (FcγRs) on immune cells, contribute to the clearance of infected cells and the modulation of antiviral immunity. Notably, Fc-mediated effector functions have been shown to correlate with protection in animal models and convalescent individuals, highlighting their importance in combating SARS-CoV-2^20–22^. In murine models deficient in functional Fcγ receptors, protection against SARS-CoV-2 was lost underscoring the essential role of FcγRs in mediating antiviral immunity^21^. The importance of Fc effector functions extends beyond SARS-CoV-2 and has been extensively studied in other viral infections, including respiratory syncytial virus (RSV), influenza, and HIV^23–25^.

Recent studies suggest that the quality of vaccine-induced antibody responses, including Fc effector functions, may decline with age^9,26^. IgG1, the predominant antibody subclass responsible for mediating ADCC, has been shown to exhibit reduced functionality in older adults^27^. Given the increasing reliance on booster doses to maintain protective immunity, understanding how aging impacts Fc-mediated responses after repeated vaccination is critical for optimizing vaccine strategies in this population.

Here, we sought to evaluate the age-dependent effects of SARS-CoV-2 vaccination on immune parameters, including neutralization, Fc effector functions, and antibody isotype-specific responses. We analyzed immune responses in a cohort of elderly individuals (mean age: 79 years; age range: 65–92 years) who had received six doses of SARS-CoV-2 vaccines. By stratifying participants into younger (≤80 years) and older (>80 years) groups, we identified age-related differences in Fc-mediated ADCC activity and IgG1 binding to the SARS-CoV-2 spike protein. These findings reveal a critical vulnerability in vaccine-induced immunity among the elderly and underscore the need for tailored approaches to enhance immune responses in this high-risk population.

## Study overview

We enrolled a cohort of 50 individuals (54% male and 46% female) who had received their sixth dose of a SARS-CoV-2 vaccine, with blood samples collected one-month post-vaccination (Table 1). The median age of the participants was 79 years, and individuals were stratified into two groups: younger (≤80 years [65–80]) and older (>80 years [81–92]). This cohort had an age range of 65–92 years old. Of this cohort, 25 individuals had the Sanofi/GSK AS03-adjuvanted (VidPrevtyn Beta) subunit vaccine, and 25 individuals had the Pfizer-BioNTech mRNA (Comirnaty Original/Omicron BA. 4–5) bivalent vaccine. This vaccine regimen represented the most common schedules among participants, although some individuals did receive mixed vaccine types across different doses. To assess vaccine-induced humoral immunity, we measured comprehensive total immunoglobulin G (IgG) responses against the SARS-CoV-2 spike (S) protein, alongside nucleocapsid (N) total IgG, using a Luminex-based flow cytometry assay. Participants with evidence of active SARS-CoV-2 infection were excluded from the study. Notably, N-specific antibody titers can decline over time, in some cases within relatively short intervals^28^ underscoring the importance of stratifying individuals based on infection status to accurately evaluate vaccine-induced immunity.

**Table 1:** Demographics of the cohort

Characteristic	*N* = 50^a^
Age	79 (64, 92)
Age Split by 80	
≤80	20 (40%)
>80	30 (60%)
Sex	
F	23 (46%)
M	27 (54%)
dose6	
mRNA	25 (50%)
Sanofi	25 (50%)

^a^Median (Q1, Q3); *n* (%).

## Comparable neutralization following six vaccine doses across age, sex, and vaccine type

**Fig. 1:**
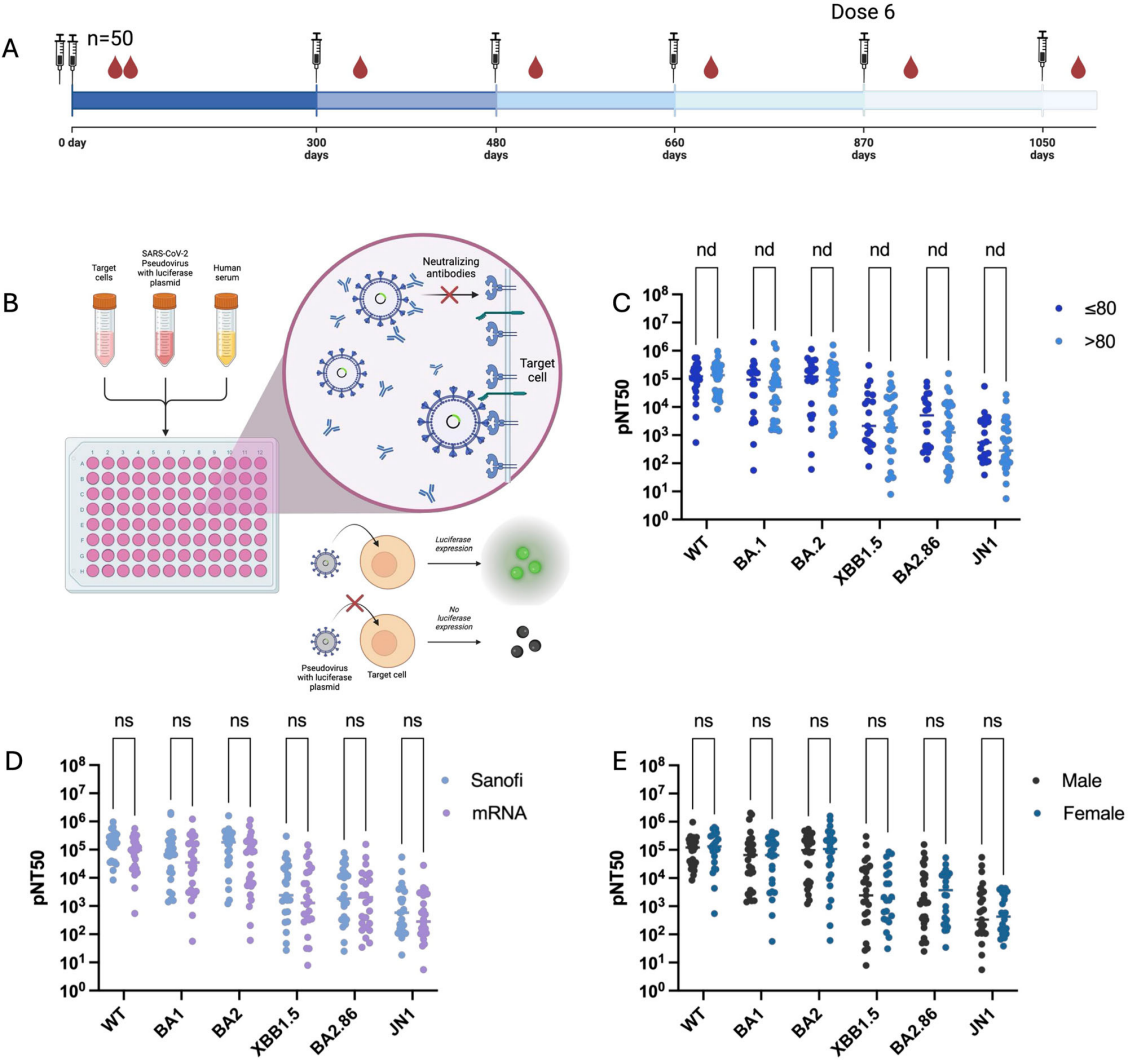
Serum neutralising antibody titres to SARS-CoV-2 variants of concern following dose 6 of COVID-19 vaccine. **A** Top, Study design. Schematic of vaccine timeline highlighting the sixth dose. Fifty-one individuals vaccinated against SARS-CoV-2 with mRNA-based vaccines and/or AZD1222 and/or sub-unit vaccine. **B** Schematic design of pseudovirus neutralization assay. **C** Neutralization titers (pNT50) stratified by age against Wu-1 D614G WT, BA.1, BA.2, XBB1.5, BA.2.86, and JN.1. **D** pNT50 stratified by vaccine-type at dose 6. **E** pNT50 stratified by sex. Multiple Mann-Whitney compare rank tests with Holm-Šídák corrections were performed to determine significance. *p < 0.05, **p < 0.005, ***p < 0.0005. NS, not significant.

To assess the impact of aging on neutralizing antibody responses, we compared neutralization titers (NT50) among younger (≤80 years) and older (>80 years) individuals following their sixth SARS-CoV-2 vaccine dose (Fig. 1A). Neutralization was evaluated against wild-type (WT) SARS-CoV-2 as well as key SARS-CoV-2 variants, including BA.1, BA.2, XBB.1.5, BA.2.86, and JN1 (Fig. 1B). Across all tested variants, we observed no significant differences in NT50 values between the two age groups, indicating that neutralizing antibody responses remained comparable at dose 6 regardless of age (Fig. 1C). This suggests that repeated vaccination may effectively preserve neutralization capacity among elderly and very elderly individuals; however, comparisons to younger cohorts (under 50 years) were not assessed in this analysis and younger individuals may show higher neutralization titers.

We further analyzed whether sex or vaccine type influenced neutralization capacity. Participants had received either mRNA-based vaccines or the Sanofi subunit vaccine, yet no significant differences in NT50 values were observed between these vaccine platforms (Figs. 1D and S1A–S1H). Similarly, neutralization responses were comparable between male and female participants, suggesting that neither sex nor vaccine formulation significantly altered the breadth or magnitude of neutralizing antibody responses at the sixth dose (Fig. 1E).

## Age-dependent decline in spike-specific IgG1 response

**Fig. 2:**
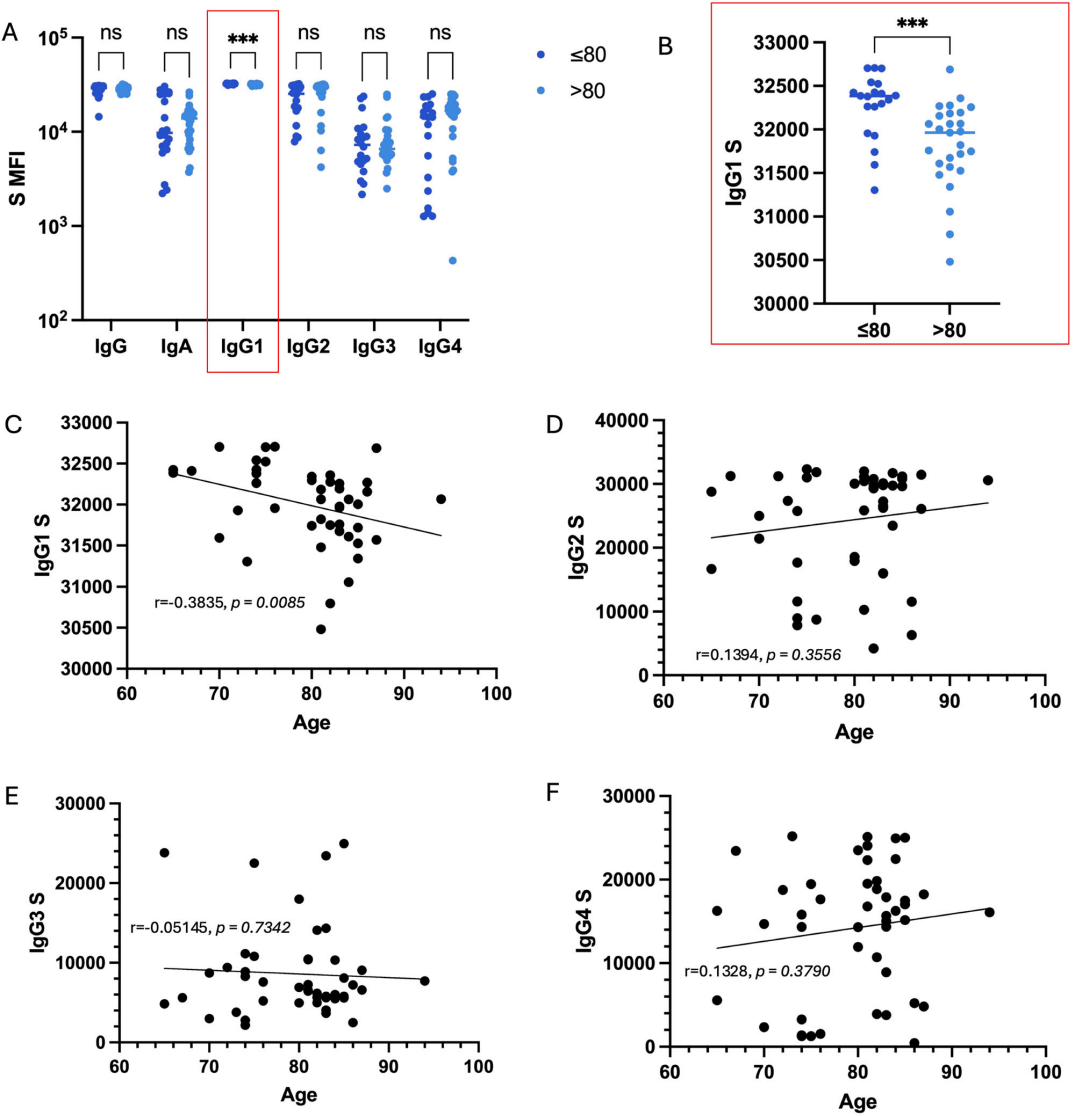
Binding serum antibody titres following dose 6 of COVID-19 vaccine. **A** Total anti-spike IgG binding antibody responses (multiple Mann-Whitney compare rank test with Holm-Šídák corrections: ***p < 0.0005). **B** Anti-spike IgG1 binding stratified by age. **C** Correlation coefficient between IgG1 S correlated with age. **D** Correlation coefficient between IgG2 S and age, non-significant. **E** Correlation IgG3 S and age, non-significant. **F** Correlation coefficient between IgG4 S and age, non-significant. Scatter plots show linear correlation line, and statistical significance was assessed by Pearson’s Correlation.

To investigate how aging affects vaccine-elicited antibody responses, we then analyzed the distribution of antibody subclasses in our cohort. Using a Luminex bead-based assay, we measured total IgG, IgA, IgG1, IgG2, IgG3, and IgG4 titers towards various regions of the wild-type and omicron SARS-CoV-2 virion including, nucleocapsid (N), omicron receptor-binding domain (RBD), RBD2, spike (S), S1, and S1 omicron (Fig. 2A). Among all subclasses, only IgG1 exhibited a significant decline in individuals aged >80 years compared to younger participants (≤80 years), suggesting a potential age-dependent reduction in the maintenance or production of this antibody subclass (Figs. 2B and S2C and S3C). In contrast, total IgG, IgA, IgG2, IgG3, and IgG4 levels against spike remained comparable between age groups, indicating a selective impairment in IgG1 response with aging (Figs. S2–S6). Similarly, when correlated with age, only IgG1 demonstrated a significant negative correlation with age (Fig. 2C–F). Importantly, anti-spike IgG3, which is the other antibody isotype known to activate ADCC, remained consistent between both age cohorts (Fig. S2E)^29^. Anti-S1 and anti-S1 Omicron IgG1 titers however, followed a similar relationship as anti-spike IgG1, as the older participants had significantly lower titers (Figs. S3C and S5C).

Given the critical role of IgG1 in engaging Fcγ receptors to activate antibody-mediated effector functions, we then sought to explore if this reduction in spike-specific IgG1 impairs the ability of antibodies to efficiently activate Fc-mediated effector functions, including ADCC.

## Decreased spike-specific IgG1 levels in elderly individuals correlate with diminished ADCC signaling activity

**Fig. 3:**
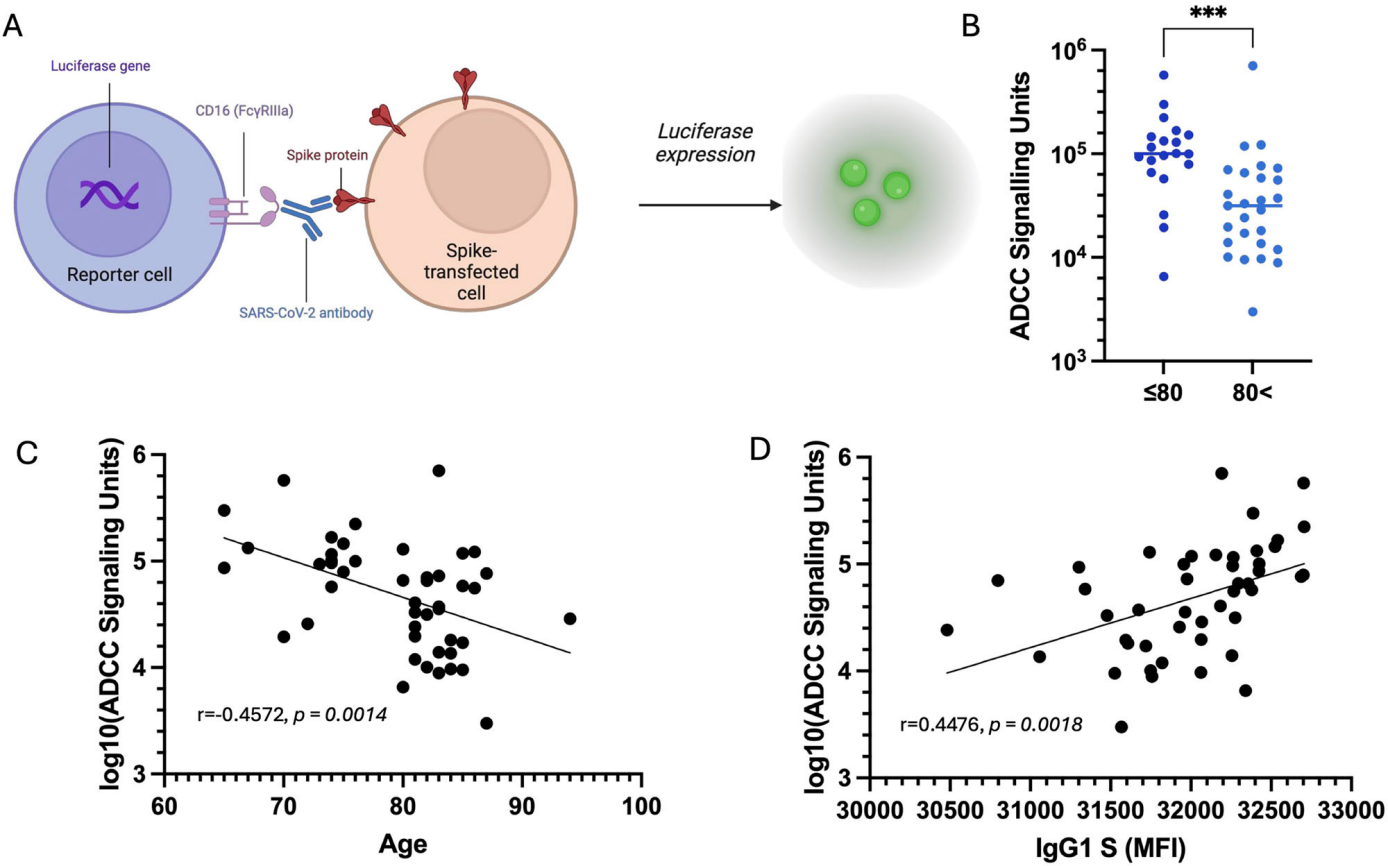
Serum antibody dependent cellular cytotoxicity activity against SARS-CoV-2 spike expressing cells following dose 6 of COVID-19 vaccine. **A** Schematic of the antibody-dependent cellular cytotoxicity (ADCC) assay. **B** Antibody-dependent cellular cytotoxicity assay stratified by age. Mann-Whitney test was used. ***p < 0.0005. **C** log(ADCC signaling units) correlated with age. **p < 0.005 . **D** Correlation coefficients between log(ADCC signaling units) and anti-spike IgG1. **p < 0.005. Scatter plots show linear correlation line, and statistical significance was assessed by Pearson’s Correlation.

To determine if the observed decline in spike-specific IgG1 levels correlated with Fc-mediated immunity, we next assessed ADCC signaling activity in our cohort. Using an ADCC signaling assay with a CD16-expressing reporter cell line, we observed a striking age-dependent decrease in ADCC activation, measured as signaling units, with individuals aged >80 years demonstrating significantly lower ADCC responses than younger participants ( ≤ 80 years), (Fig. 3A, B). This disparity suggests that, despite comparable neutralizing antibody titers, older individuals may have diminished Fc-mediated effector functions, potentially limiting their ability to eliminate virus-infected cells.

To further explore the relationship between ADCC and age, we conducted a correlation analysis between spike-specific ADCC signaling and age. We observed a significant negative correlation between age and ADCC signaling, reinforcing the notion that aging is associated with a progressive decline in Fc-mediated effector function (Fig. 3C). Notably, the age-dependent decline in IgG1 levels closely mirrored the observed reduction in ADCC activity, and correlation analysis revealed a significant positive association between spike-specific IgG1 and ADCC signaling (Fig. 3D). This finding is consistent with the well-established role of IgG1 in facilitating ADCC through engagement of Fcγ receptors on effector cells. The concordant decrease in both IgG1 and ADCC signaling suggests that diminished IgG1 responses in elderly individuals may underlie their impaired Fc-mediated immune function.

These findings suggest that while neutralization remains intact with repeated vaccination, the capacity for ADCC-mediated immune clearance may be compromised in the elderly, underscoring the need for vaccine strategies that enhance Fc effector functionality in this vulnerable population. Given the importance of ADCC in viral clearance and vaccine-mediated protection, these results highlight a potential vulnerability in the immune response of older adults. Tailoring vaccine strategies to enhance IgG1 production may therefore be a critical approach for optimizing immunity in this population, particularly in the context of booster vaccination.

## Implications for age-associated ADCC defects

Our findings reveal a key deficiency in vaccine-induced immunity among elderly individuals, with potential implications for future vaccine design, particularly for those at highest risk of severe COVID-19 outcomes. Despite maintaining comparable neutralization capabilities, older individuals exhibit significantly lower levels of spike-specific IgG1, which likely contributes to the observed reduction in ADCC responses, an essential mechanism for clearing infected cells, controlling viral spread, and playing a role in modulating disease severity. While neutralization activity remains consistent between age groups, the ability to neutralize emerging SARS-CoV-2 variants, such as XBB1.5 and JN.1, remains suboptimal, potentially increasing susceptibility to breakthrough infections. Interestingly, the bivalent mRNA and adjuvanted subunit vaccines elicited similar levels of neutralization, which we hypothesize may be due to prior infection (imprinting) or heterologous epitope boosting, however more in-depth analysis would be required to prove this. Given that IgG1 plays a critical role in Fc-mediated effector functions, its diminished presence in older adults may impair the antibody-mediated activation of FcγRIIIa (CD16) on effector cells, thereby reducing their capacity to eliminate virally-infected cells^17,30,31^. This age-related decline in IgG1-mediated immunity may reflect broader immunosenescence patterns, including both qualitative and quantitative shifts in humoral immunity, with implications for protection against severe disease and death^32–34^. These findings underscore the importance of tailoring vaccine strategies for elderly populations, ensuring that immunization approaches not only elicit strong neutralizing antibody responses, but also antibodies that enable Fc-mediated effector functions, critical for comprehensive disease protection. Whether this reduction in IgG1 reflects a diminished capacity to produce spike-specific antibodies that mediate cellular immunity or an overall shift in isotype or antigen-specificity distribution warrants further investigation.

Importantly, this age-dependent immune decline is not unique to SARS-CoV-2 vaccination. Similar declines in IgG have been reported in the context of pneumococcal disease with elderly individuals demonstrating lower total antibody titers and a reduction in IgG1 compared to younger adults^35,36^. Likewise, influenza is particularly severe among people aged more than 65 years, with elderly accounting for 90% of all influenza-related deaths outside of pandemic seasons^37,38^. It is possible that the increase in influenza-related mortality among elderly individuals is related to the impaired antibody response, and corresponding blunted Fc-mediated immunity^39^. This decline in IgG1 likely plays a role in impaired Fc-mediated effector functions, including ADCC, which is critical for the clearance of virus-infected cells, as IgG1 and IgG3 are the main isotypes for mediating ADCC^29^. Although the direct link between reduced IgG1 production and increased influenza mortality in the elderly has not been firmly established, the decline in IgG1 and corresponding impaired ADCC response could be a contributing factor to higher influenza-related mortality. Given the established role of ADCC in influencing influenza disease severity and enhancing viral clearance, an impairment in the SARS-CoV-2 setting could indicate a broader deficiency in antibody-mediated effector function with aging.

## Aging and B cell related changes

One possible explanation for the lower IgG1 levels in elderly individuals is a decline in class-switching efficiency, reduced production of B and T cells in the bone marrow, and diminished function of mature lymphocytes^40–42^. Alternatively, it is possible that elderly individuals produce similar amounts of total IgG but exhibit skewed subclass distribution, favoring isotypes such as IgG2 or IgG4 that do not induce ADCC-activation^29,43^. The extent to which this altered subclass balance affects other Fc-mediated immune functions beyond ADCC, such as antibody-dependent cellular phagocytosis (ADCP) or complement activation, remains of interest.

Another potential contributor to the reduced spike-specific IgG1 levels and impaired ADCC responses in elderly individuals is clonal hematopoiesis (CH), a phenomenon common with aging. CH, particularly involving lymphoid-biased clones, can influence B cell repertoire, diversity, and function^44–46^. As B cell development is highly regulated by clonal selection processes, the expansion of certain clones at the expense of others may lead to an altered antibody profile. If elderly individuals exhibit increased clonal expansions of B cells that produce non-specific, lower-affinity, or functionally impaired antibodies, this could potentially lead to reduced production of high-quality antigen-specific IgG1 post-vaccination. Furthermore, CH-associated mutations can also be involved in immune cell signaling and inflammatory states, which may skew B cell differentiation pathways away from optimal IgG subclass switching^47,48^, as antibody isotype switching is largely mediated by cytokine expression^49–51^. Investigating the intersection of CH and vaccine-induced immunity may provide crucial insights into age-related immune responses and strategies to enhance these antibody responses in older adults.

Similar to CH, age-associated B cells (ABCs), or atypical B cells, have been demonstrated to accumulate with increasing age and may contribute to reduced production of ADCC-activating antibodies^52–54^. These cells are characterized by higher expression of the inhibitory FcγRIIB receptor, which is known to impair B cell activation and function^55–58^. Similarly, individuals that present with higher baseline percentages of ABCs have correlated with decreased levels of antigen-specific memory B cells and reduced antigen availability for immune stimulation^58^. This mechanism may contribute to the diminished vaccine responses in elderly individuals, including lower spike-specific IgG1 levels and impaired ADCC responses. Likewise, studies have linked the expansion of ABCs to chronic antigen exposure and inflammation, both of which are more prevent in aging populations, further supporting their potential role in age-associated immune dysfunction^59–61^.

Understanding the mechanisms underlying the reduced ADCC observed in our cohort is critical in developing methods and approaches to better protect elderly populations from severe COVID-19. If the deficit is driven by impaired IgG1 production, enhancing germinal center responses through adjuvants or tailored booster formulations may help restore ADCC activity. However, if this decline is rooted in effector cell dysfunction and decreasing NK cell populations or functionality, focusing solely on enhancing antibody responses may be insufficient. Addressing this gap requires further studies evaluating Fc-mediated antibody responses alongside effector cell functionality in elderly populations. These insights will be essential in the development of strategies to prevent severe COVID-19 in the elderly.

This study has limitations that should be considered when interpreting the findings. Firstly, the sample size was relatively small, which may limit the generalizability of our results. Likewise, we recognize unequal distribution of vaccine types across age groups. However, we consistently observed lower ADCC signaling with increasing age, regardless of vaccine type. In addition, we did not assay spike specific B cells to explore whether there was an atypical phenotype or other abnormality in the elderly group. Additionally, the cross-sectional nature of the study prevents us from assessing longitudinal changes in ADCC over time as other timepoints were unavailable for analysis. Future studies with longitudinal design and pre-vaccination baseline serum could clarify how these immune responses evolve upon repeated SARS-CoV-2 exposure or vaccination. Unfortunately, PCR-confirmed infection data and hospitalization records were not available for all individuals, limiting our ability to interpret the results in the context of prior infection. Second, we used a lentiviral-based pseudotyped virus system rather than live SARS-CoV-2 to evaluate neutralization and viral entry^62^. While pseudoviruses are well-established tools for studying viral entry and antibody-mediated neutralization, they may not fully replicate the complexities of infection with replication-competent virus. Additionally, ADCC was assessed using a surrogate reporter assay rather than cytotoxicity assays with live autologous NK cells. However, previous studies have demonstrated that this surrogate assay strongly correlates with functional ADCC responses^13,63,64^. Despite these limitations, our study provides important insights into the age-dependent differences in immunity following repeated vaccination. Importantly, these findings may reflect both vaccine response differences and age-associated immunoglobulin shifts.

## Methods

### Study population details

Eligible individuals included community participants who had received six doses of a SARS-CoV-2 vaccine and provided blood samples at 3 weeks following vaccination. Participants were categorized by age into two groups (≤80 and >80 years) to assess the impact of aging on vaccine-elicited immune responses. The primary outcome of interest was ADCC activity, measured by a surrogate reporter assay that quantifies FcγRIIIa-mediated signaling. Secondary outcomes included antibody antigen and isotype analysis and serum neutralization activity against SARS-CoV-2 variants. Pseudovirus assays were employed to measure serum neutralization. All participants provided written informed consent, and the study was conducted in accordance with institutional ethical guidelines.

### Ethical approval

This study was conducted in accordance with the ethical principles outlined in the Declaration of Helsinki. The study was approved by the East of England-Cambridge Central Research Ethics Committee (REC ref. ^17^: /EE/0025). The participants provided necessary informed consent. For studies involving human serum samples, appropriate biosafety protocols were followed to ensure safe handling and processing. All pseudovirus-based assays were performed under biosafety level 2 (BSL-2) conditions, in compliance with institutional and national guidelines. All data were anonymized to protect participant confidentiality.

### Statistical analyses

Statistical analyses were conducting using GraphPad Prism software (version 10.4.1). Measurements were performed in duplicate and were analyzed via Glomax luminometer. All statistical tests are described in figure legends. Linear regressions were performed to model the association between the various parameters. Statistical analyses for the linear regressions were also performed on Prism GraphPad.

### Generation of pseudotyped viruses

Viral entry of SARS-CoV-2 wild-type (WT) and Omicron-descendent variants in the presence of host neutralizing antibodies was assessed using lentivirus-based pseudotyped viruses expressing luciferase. This system mimics replication-competent SARS-CoV-2 infection without requiring high-level biosafety containment. Pseudotyped viruses were generated by co-transfecting HEK 293 T cells with 11 μL of Fugene®HD Transfection Reagent (Promega), 1 μg of an HIV-1 Gag-Pol-Tat-Rev packaging vector (p8.91), 1.5 μg of a firefly luciferase reporter construct (pCSFLW), and 1 μg of a SARS-CoV-2 spike expression plasmid (pcDNA3.1) encoding the variant of interest. These plasmids contained codon-optimized SARS-CoV-2 spike sequences with a 19-amino acid C-terminal deletion (Δ19) to enhance spike protein expression on pseudotyped particles. Following transfection, HEK 293 T cells were incubated at 37 °C with 10% CO₂. Viral supernatant was collected at 48 and 72 hours post-transfection, filtered (0.45 μM), and stored at −80 °C. The infectious dose of pseudotyped virus was determined using the Bright-Glo™ Luciferase Assay System (Promega) and quantified with a GloMax® Navigator Microplate Luminometer (Promega).

### Cell lines and culture conditions

HEK 293 T CRL-3216 cells, which express the SV40 T-antigen, were used to produce pseudotyped virus. HeLa-ACE2 cells, stably transduced with the ACE2 receptor, served as target cells for measuring serum neutralizing antibody titers against pseudotyped virus entry. All cells were cultured at 37 °C in DMEM supplemented with 10% fetal calf serum, 2 mM glutamine, 100 U/mL penicillin, and 0.1 mg/mL streptomycin.

### Serum pseudotype neutralization assay

Neutralization assays were performed using HeLa cells stably expressing human ACE2 to assess the functional capacity of vaccine-induced antibodies. Pseudotyped SARS-CoV-2 spike virus neutralization assays were performed to evaluate functional antibody responses, as these assays have been shown to closely correlate with neutralization tests using fully infectious wild-type SARS-CoV-2^62,65^. SARS-CoV-2 spike-pseudotyped lentiviral particles encoding luciferase were incubated with serial dilutions of heat-inactivated vaccinee sera in duplicate for 1 h at 37 °C^66^. Cell-only and virus-plus-cell controls were included to establish baseline luminescence. Following incubation, HeLa ACE2-expressing cells were added to each well, and plates were maintained at 37 °C in a 5% CO₂ environment for 48 h. Luminescence was then quantified using the Bright-Glo Luciferase Assay System (Promega, UK) to determine neutralization activity. Neutralization was calculated relative to virus-plus-cell controls, and 50% neutralizing titer (NT₅₀) values were derived using GraphPad Prism. The limit of detection for neutralization was set at an NT₅₀ of 20, and within each group, NT₅₀ values were summarized as geometric mean titers (GMT). Nonlinear regression method, [inhibitor] vs. normalized response variable slope in GraphPad Prism was used. Statistical comparisons between groups were conducted using the Wilcoxon signed-rank test or the Mann-Whitney U test with Holm-Šídák corrections. Spearman’s correlation matrix was generated via R v4.4.1^67^ using Hmisc v5.2.0^68^ and visualized using corrplot v0.95^69^.

### Multiplex particle-based flow cytometry (Luminex) for SARS-CoV-2 serological analysis

Recombinant SARS-CoV-2 nucleocapsid (N), spike (S), and receptor-binding domain (RBD) proteins were covalently conjugated to distinct carboxylated bead sets (Luminex, Netherlands) to generate a multiplexed serological assay. Antibody binding was assessed using a Luminex-based flow cytometry platform, as previously described^70^. Positive and negative controls were used to establish background readings. Specific binding was quantified and reported as mean fluorescence intensity (MFI).

### Antibody-dependent cell-mediated cytotoxicity response through FcɣRIIIa (CD16) signaling assay

To evaluate the ability of antibodies to engage spike-expressing cells and activate FcɣRIIIa (CD16) signaling, we utilized a luminescence-based ADCC reporter assay^13^. HEK 293 T cells were plated at a density of 2 × 10⁴ cells per well in white, tissue culture-treated 96-well plates and incubated for 4 h at 37 °C with 5% CO₂. Cells were then transiently transfected with 50 ng/well of a SARS-CoV-2 spike expression plasmid using Fugene® HD Transfection Reagent (Promega) and maintained under the same conditions for 48 h. On the day of the assay, the culture media was replaced with 25 μL of assay media (RPMI 1640 supplemented with 4% low IgG FBS), and plates were returned to the incubator. Fixed heat-inactivated serum dilution samples (final dilution 1:90) or monoclonal antibodies (30 ng/μL) were prepared in assay media and added to the transfected cells, followed by a 1-hour incubation at 37 °C with 5% CO₂. Each sample was assessed in two independent experiments, with two technical replicates per experiment. After incubation, Jurkat-Lucia NFAT-CD16 reporter cells (Promega) were introduced at 75,000 cells per well and co-cultured for 18 h. Luminescence was then detected using the Bright-Glo™ Luciferase Assay System (Promega) and quantified with a GloMax® Navigator Microplate Luminometer (Promega). Relative luminescence units (RLU) were adjusted by subtracting background signals from “media-only” and “cell-only” controls. Each plate included Sotrovimab (GlaxoSmithKline) as a positive control, along with two seropositive plasma samples—one with high ADCC activity and one with low ADCC activity. Additional controls comprised untransfected cells, transfected cells, and negative controls (media-only and cell-only conditions) to ensure consistent normalization across all assay plates.

## Supplementary information

NPJ_FiguresSUPPS

## Data Availability

Raw data from this study are available upon request from the corresponding author. This paper does not report the generation of any original code. The data required to evaluate the conclusions in the paper are present in the text and/or the supplementary materials.
