# Supplementary material for: Age-associated defect in ADCC response to COVID-19 vaccine
Source: NPJ Vaccines. 2025 Jul 1;10:132. doi: 10.1038/s41541-025-01196-9 (PMC12217920; doi:10.1038/s41541-025-01196-9)
Supplement: Supplementary file 1 — NPJ_FiguresSUPPS [file 41541_2025_1196_MOESM1_ESM.pdf]

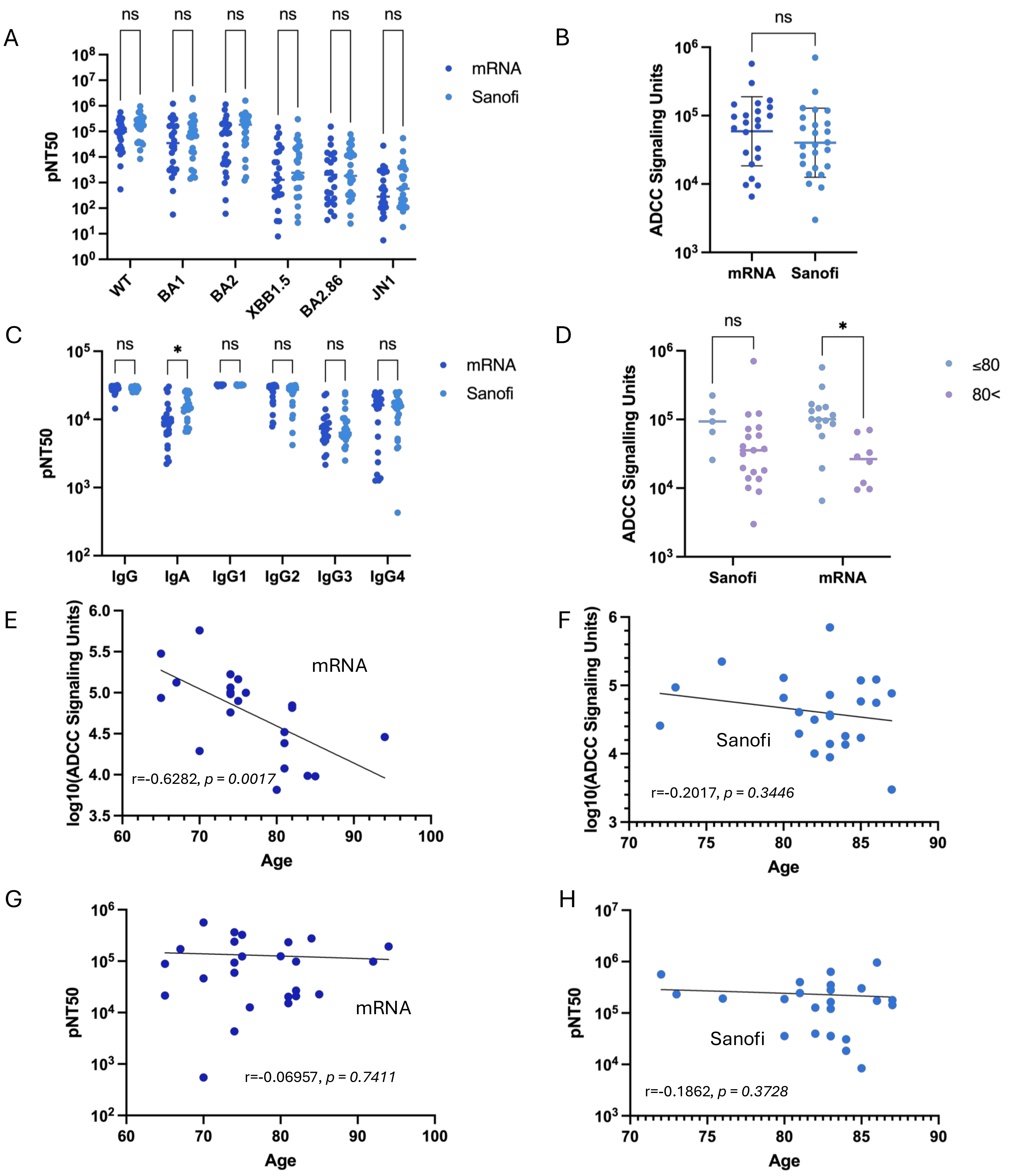

Figure S1. **a,** Neutralization titers (pNT50) stratified by vaccine type against Wu-1 D614G WT, BA.1, BA.2, XBB1.5, BA.2.86, and JN.1. **b**, Antibody-dependent cellular cytotoxicity assay stratified by vaccine type. **c,** Total anti-spike IgG binding antibody responses. Multiple Mann-Whitney tests were performed. *$p<0.05$. **d,** Antibody-dependent cellular cytotoxicity assay stratified by vaccine type and age. Multiple Mann-Whitney tests were performed. *$p<0.05$. **e,** mRNA log(ADCC signaling units) correlated with age. **$p<0.005$ **f,** Sanofi log(ADCC signaling units) correlated with age. **g,** mRNA WT pNT50 correlated with age. **h,** Sanofi WT pNT50 correlated with age. Scatter plots show linear correlation line, and statistical significance was assessed by Pearson's Correlation.

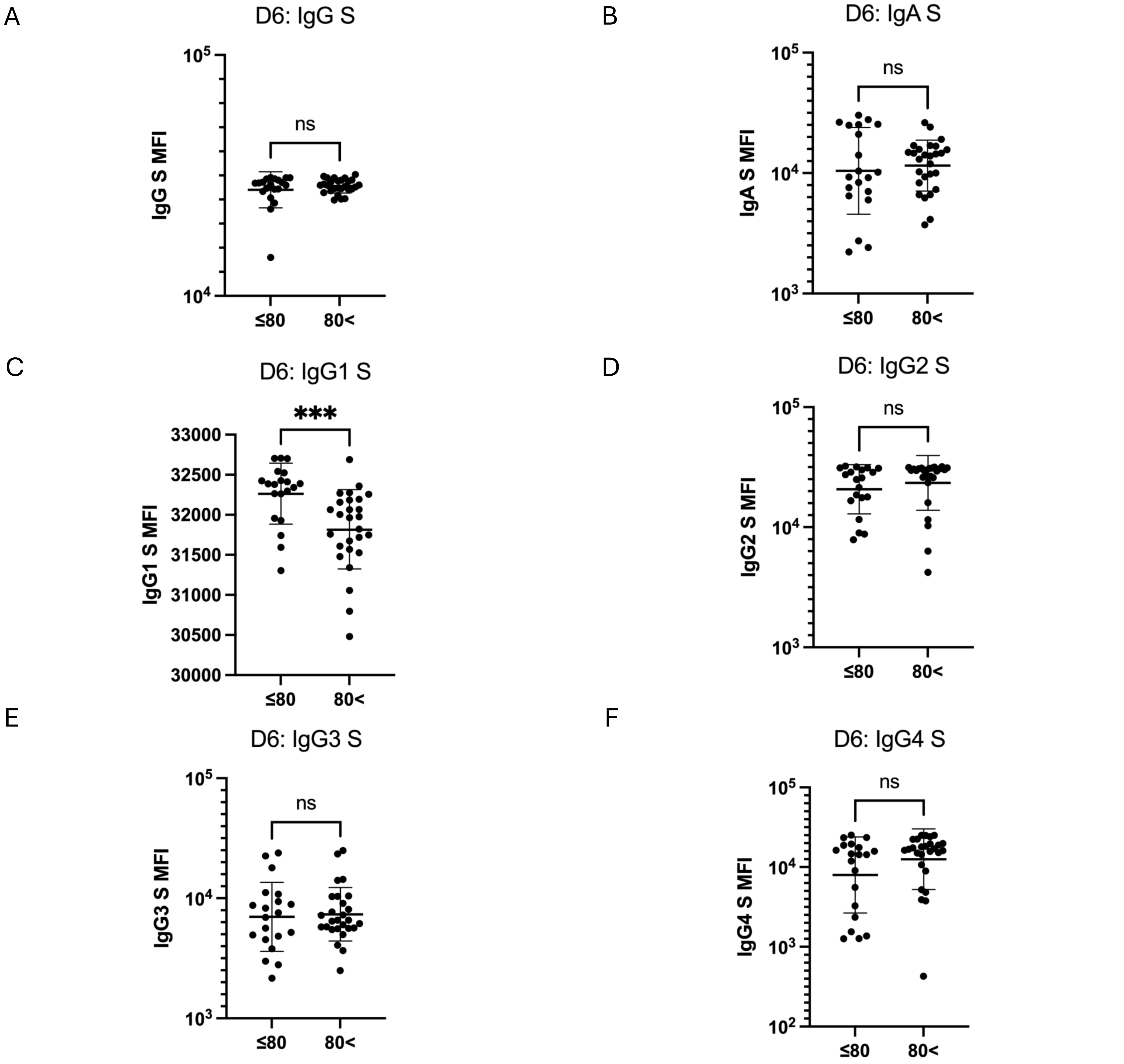

Figure S2. **a,** Total anti-spike IgG binding antibody responses. **b**, anti-spike IgA binding antibody responses. **c,** anti-spike IgG1 binding antibody responses. ****p*<0.0005. **d** anti-spike IgG2 binding antibody responses. **e,** anti-spike IgG3 binding antibody responses. **f,** anti-spike IgG4 binding antibody responses. Mann-Whitney tests were performed on all analyses. ****p*<0.0005.

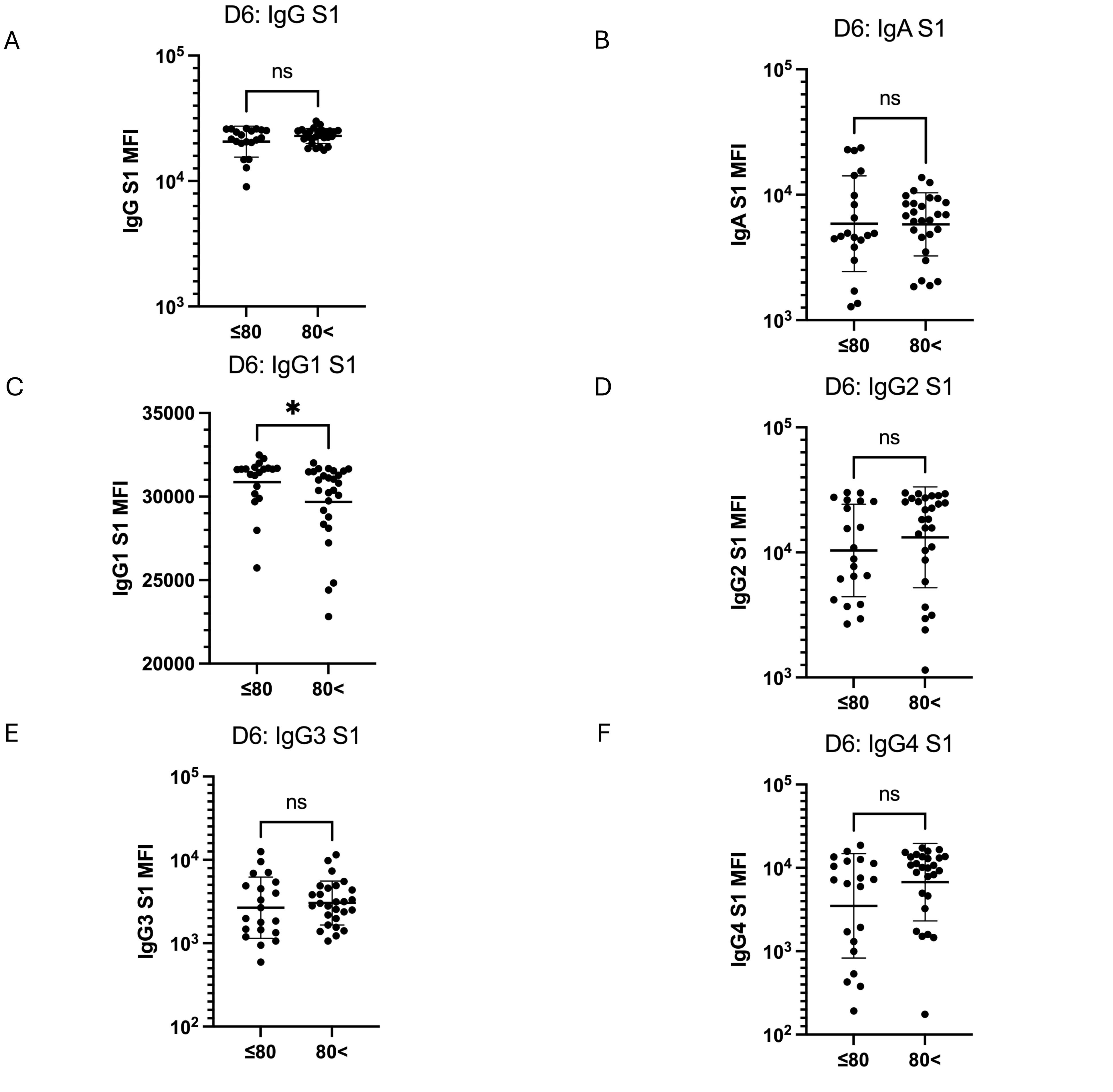

Figure S3. **a,** Total anti-S1 IgG binding antibody responses. **b**, anti-S1 IgA binding antibody responses. **c,** anti-S1 IgG1 binding antibody responses. *p<0.05. **d** anti-S1 IgG2 binding antibody responses. **e,** anti-S1 IgG3 binding antibody responses. **f,** anti-S1 IgG4 binding antibody responses. Mann-Whitney tests were performed. *p<0.05.

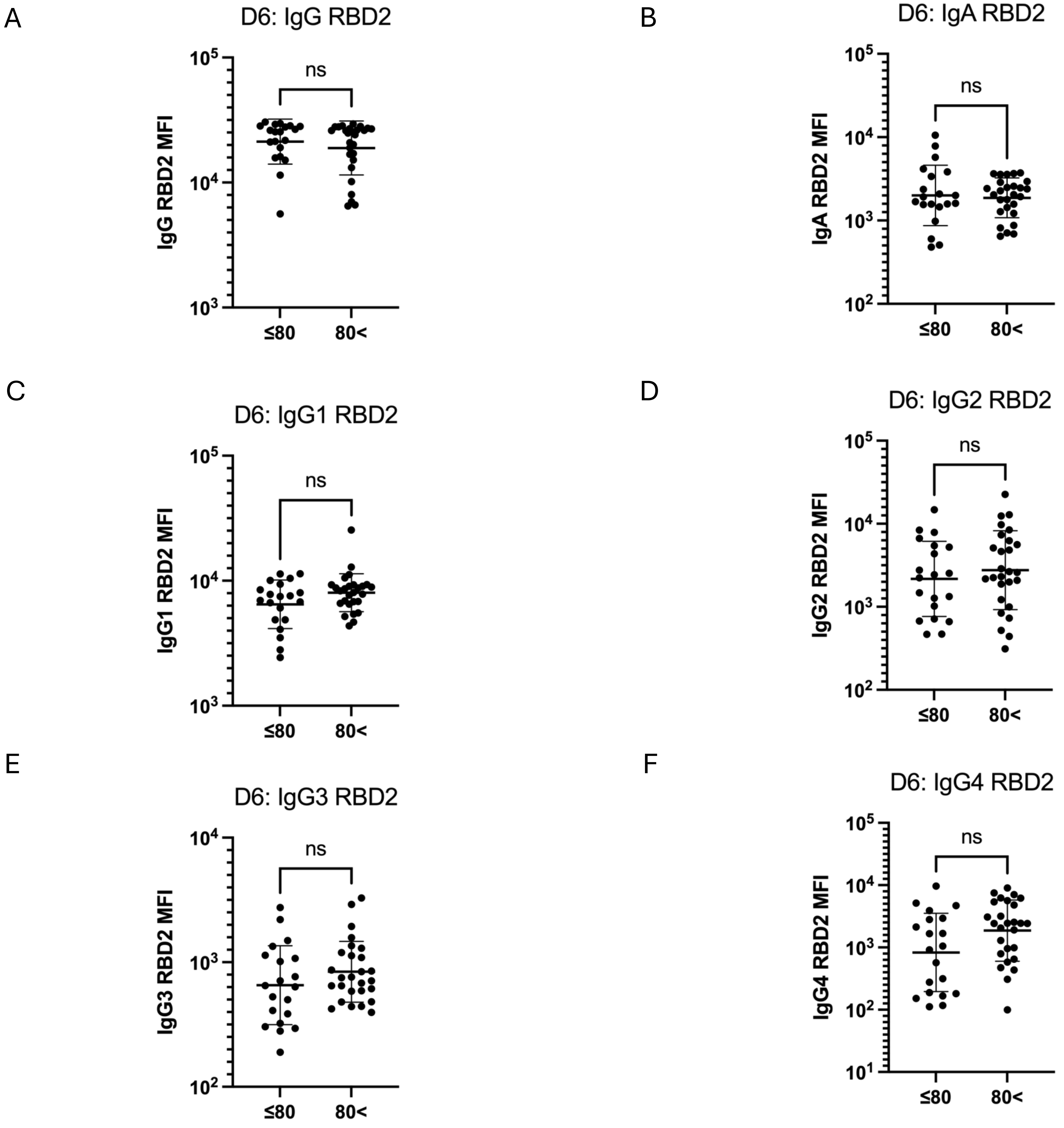

Figure S4. **a,** Total anti-RBD2 IgG binding antibody responses. **b**, anti-RBD2 binding antibody responses. **c,** anti-RBD2 IgG1 binding antibody responses. **d** anti-RBD2 IgG2 binding antibody responses. **e,** anti-RBD2 IgG3 binding antibody responses. **f,** anti-RBD2 IgG4 binding antibody responses. Mann-Whitney tests were performed.

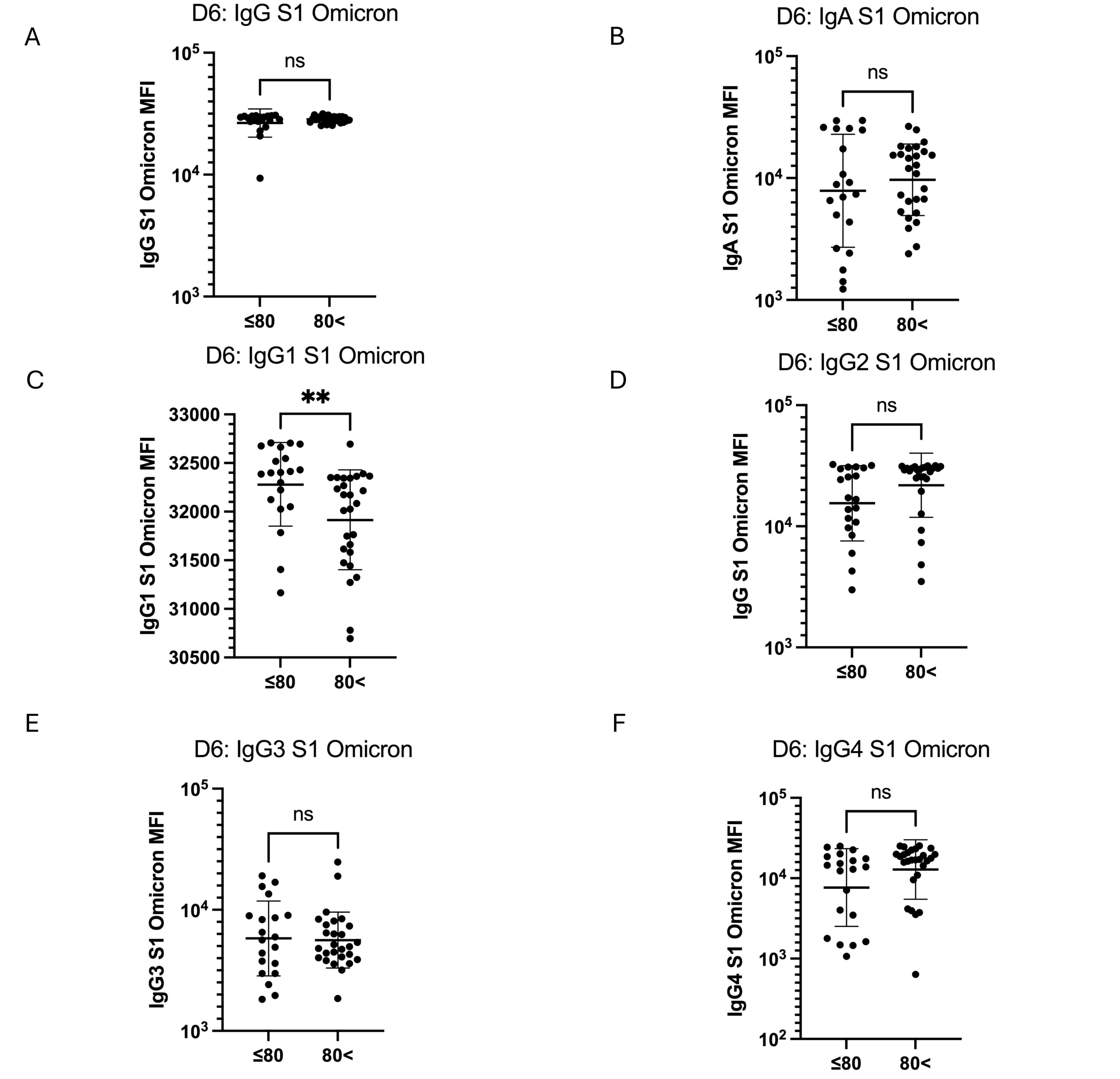

Figure S5. **a,** Total anti-S1 Omicron IgG binding antibody responses. **b**, anti-S1 Omicron binding antibody responses. **c,** anti-S1 Omicron IgG1 binding antibody responses. $^{**}p<0.005$. **d** anti-S1 Omicron IgG2 binding antibody responses. **e,** anti-S1 Omicron 2 IgG3 binding antibody responses. **f,** anti-S1 Omicron IgG4 binding antibody responses. Mann-Whitney tests were performed. $^{**}p<0.005$.

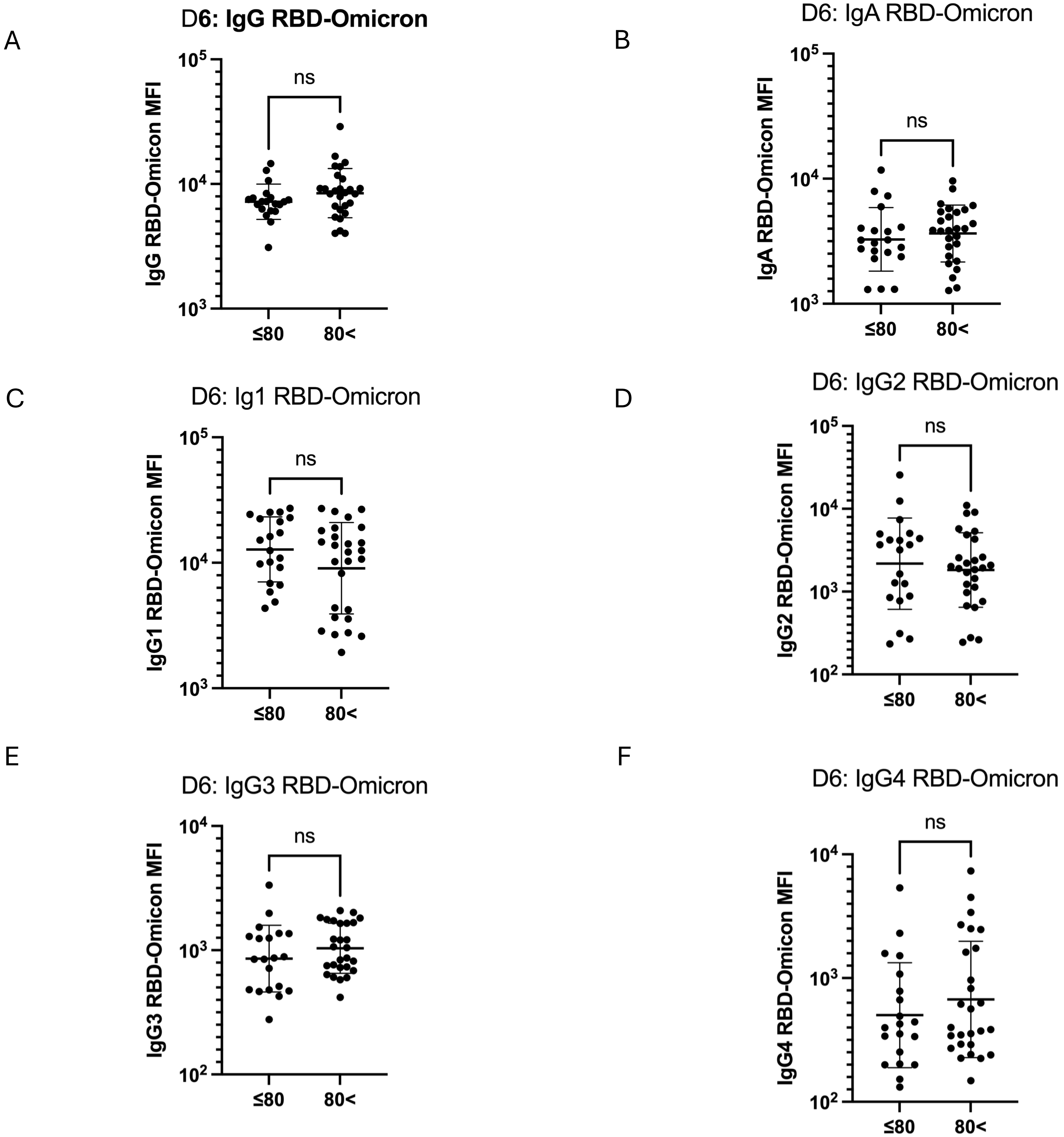

Figure S6. **a,** Total anti-RBD Omicron IgG binding antibody responses. **b**, anti-RBD Omicron binding antibody responses. **c,** anti-RBD Omicron IgG1 binding antibody responses. **d** anti-RBD Omicron IgG2 binding antibody responses. **e,** anti-RBD Omicron IgG3 binding antibody responses. **f,** anti-RBD Omicron IgG4 binding antibody responses. Mann-Whitney tests were performed.

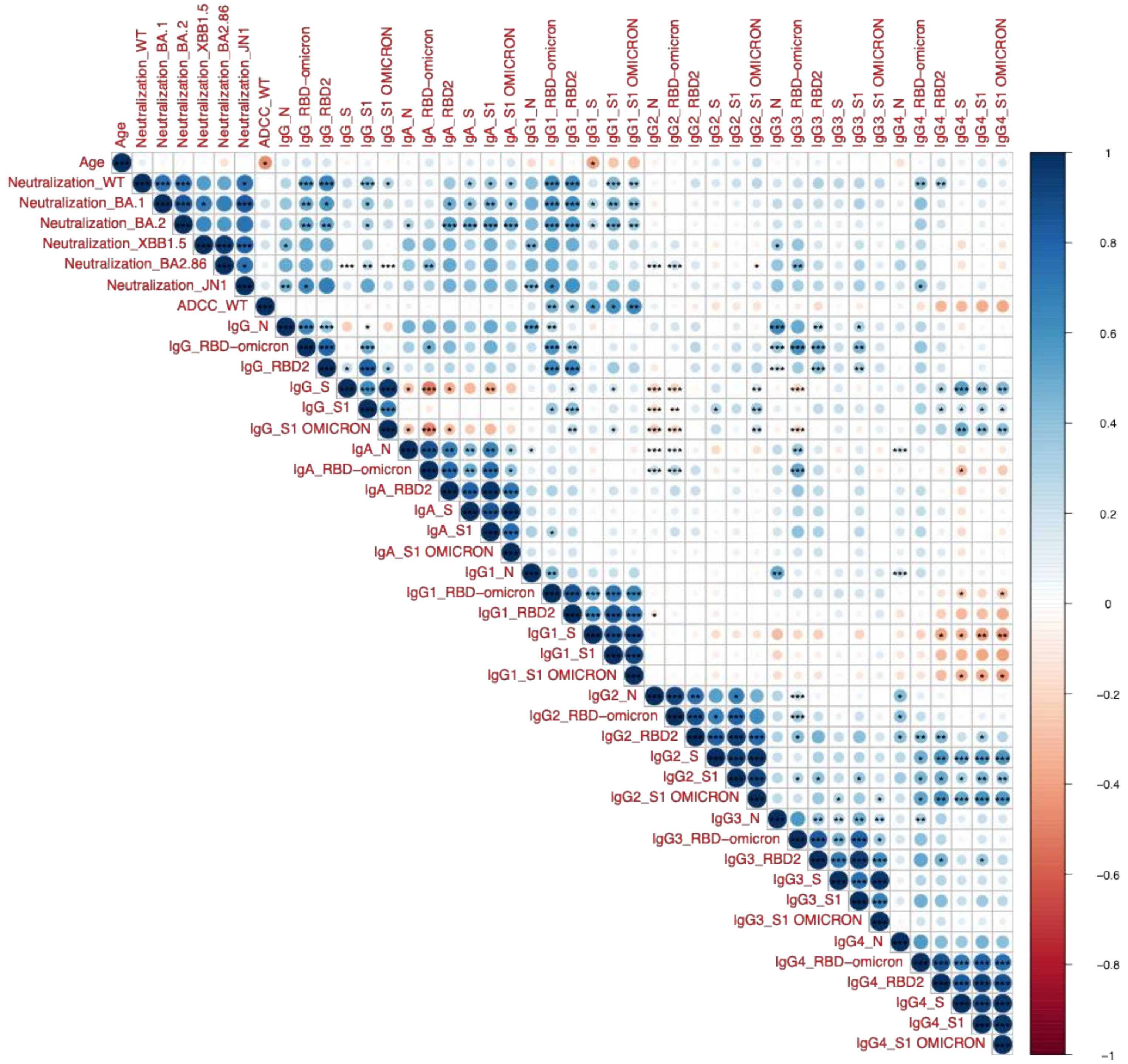

Figure S7. Spearman's correlation matrix of Neutralization, ADCC, and IgG subunit binding. Color scale represents the strength of correlation, positive correlations are blue, negative correlations are red, and strength of correlations is represented by color darkness. *p* *<0.05;**<0.01;***<0.001.
